# The Local Heroes Project: a youth-led pan-India hyperlocal crisis relief model during the COVID-19 pandemic

**DOI:** 10.3389/fpubh.2023.1282289

**Published:** 2023-12-07

**Authors:** Dhrumil Patil, Priyansh Shah, Shirish Rao, Anoushka Arora, Devarsh Shah, Pratik Sarangi, Krittika Gogoi, Manaswi Dutta, Yashsri Thakore, Srushti Pawar, Zhenyu Zhang, Mamta Swaroop

**Affiliations:** ^1^World Youth Heart Federation, Vadodara, India; ^2^Beth Israel Deaconess Medical Center, Harvard University, Boston, MA, United States; ^3^Seth G.S. Medical College and K.E.M. Hospital, Mumbai, India; ^4^Smt. Nathiba Hargovandas Lakhmichand Municipal Medical College, Ahmedabad, India; ^5^Baroda Medical College, Vadodara, India; ^6^Vardhman Mahavir Medical College and Safdarjung Hospital, Delhi, India; ^7^Assam Medical College, Dibrugarh, India; ^8^Gauhati Medical College, Guwahati, India; ^9^Zydus Medical College and Hospital, Dahod, India; ^10^SMBT Institute of Medical Sciences and Research Centre, Igatpuri, India; ^11^Department of Cardiovascular Sciences, Hypertension and Cardiovascular Epidemiology, KU Leuven, Leuven, Belgium; ^12^Kern Medical Center, Bakersfield, CA, United States

**Keywords:** crisis relief, youth, volunteers, local, COVID-19, donation

## Abstract

**Introduction:**

There was shortage of essential diagnostic and therapeutic supplies in public hospitals during the second wave of COVID-19 in India.

**Materials and methods:**

The Local Heroes Project, a hyperlocal project initiated by the World Youth Heart Federation (WYHF). Pilot project was conducted in six cities, and a nationwide project was scaled up to 58 city groups with 438 volunteers. Three-step model of needs assessment, fundraising, and establishment of the supply chain was undertaken. A national team was formed consisting of representatives from multiple international organizations and stakeholders. Local Volunteers were recruited and empowered in each city to conduct donation drives. The Qualitative Comparative Analysis (QCA) model was used to assess the impact of the intervention.

**Results:**

48.2% of the city groups completed needs assessment and 37.9% completed their donations. Factors such as team strength more than 4, local needs assessment, regular reporting during monthly meeting, receptive local administration, donation to more than 2 health centers and donation of supplies worth >= Rs 5,000 in each city (raw coverage 0.44, consistency 1) were more important contributors for success of the outcome. Supplies worth INR 2.45 million were donated.

**Conclusion:**

Hyperlocal projects can effectively address essential supply shortages. A three-step model of needs assessment, fundraising, and supply chain establishment can be an effective approach. Community involvement and donations are crucial for the success and sustainability of such projects.

## 1 Introduction

India experienced a massive surge of COVID-19 cases and deaths during the second wave of the COVID-19 pandemic that hit in the middle of March 2021 (1). There are many plausible reasons for the increase in fatalities of the second wave, such as various mutating strains leading to increased infectivity, complacency on masking and quarantining protocols, and delayed initiation of the vaccine drive (2). During the time of crisis, the majority of the general population relied on already overburdened public hospitals with scarce resources. A rapidly increasing number of cases accelerated the depletion of essential medications (3). The second wave lasted for 99 days, peaking on April 23rd 2021 with 17,937 single-day new cases (4). The second wave is considered to be far more severe than the first wave India saw in 2020 (5). Frontline workers across India were overworked, hospitals understaffed and the healthcare system overwhelmed. This view was shared by the frontline workers of the entire country (6).

World Youth Heart Federation (WYHF), a non-profit organization, which provides a dynamic platform to individuals below the age of 35 years to work towards improving the cardiovascular health of the society undertook an initiative to tackle the crisis in the availability of resources. Volunteers of WYHF working at the frontline in Vadodara district witnessed an increase in the number of preventable deaths due to pulmonary embolism, if there had been an adequate supply of heparin. While there was a significant interest in donating oxygen supplies, which had a positive impact, the scarcity of medications and personal protective equipment (PPE) did not receive nearly enough attention (7). The scarcity was the result of a demand–supply mismatch which led to pharmaceutical companies increasing prices. This adversely impacted the ability of government hospitals to purchase the medicines and PPEs that they needed. The importance of a rapid needs assessment of public hospitals became clear. Youth volunteers were mobilized to raise funds during the resource crisis to facilitate procurement of essential drugs and PPEs and thus bridge these gaps during the COVID-19 pandemic. These volunteers worked locally in multiple cities across India, and were called “Local heroes,” and the initiative was called “The Local Heroes Project (LHP)”.

In this article we assess the impact of the Local Heroes Project, whose primary outcome was to supply essential medications and PPEs to healthcare centers with shortage, via local fundraising and resource mobilization by the local youth.

## 2 Materials and methods

### 2.1 Intervention

The Project was first initiated in the city of Vadodara. A list of deficient essential supplies was provided to Volunteers of WYHF by the superintendent of the government hospital. To assess the resource limitations in other regions, WYHF members in five other cities were contacted. This revealed the existence of unmet needs even in those locations. A pilot project was established by recruiting volunteers in the regions of Vadodara, Delhi, Agra, Jammu, Goa, and Surat. The core team of WYHF recruited volunteers by sending out flyers and registration forms via social media channels and personal contacts. Each city team consisted of 3 to 12 volunteers, a fundraising lead, procurement lead, and a team lead. Separate WhatsApp groups were created for each team which was supervised and coordinated by a WYHF core member. A three-step model of (1) needs assessment, (2) fundraising, and (3) establishment of supply chain, was developed. Donations were initially made to the tertiary care hospital of Vadodara district (Sir Sayajirao General Hospital) followed by 2 rural primary health centers (PHCs) each, in the cities of Surat, Delhi, Jammu and Goa. This was achieved with the assistance of public health departments of the medical colleges and medical officers.

**Figure 1:**
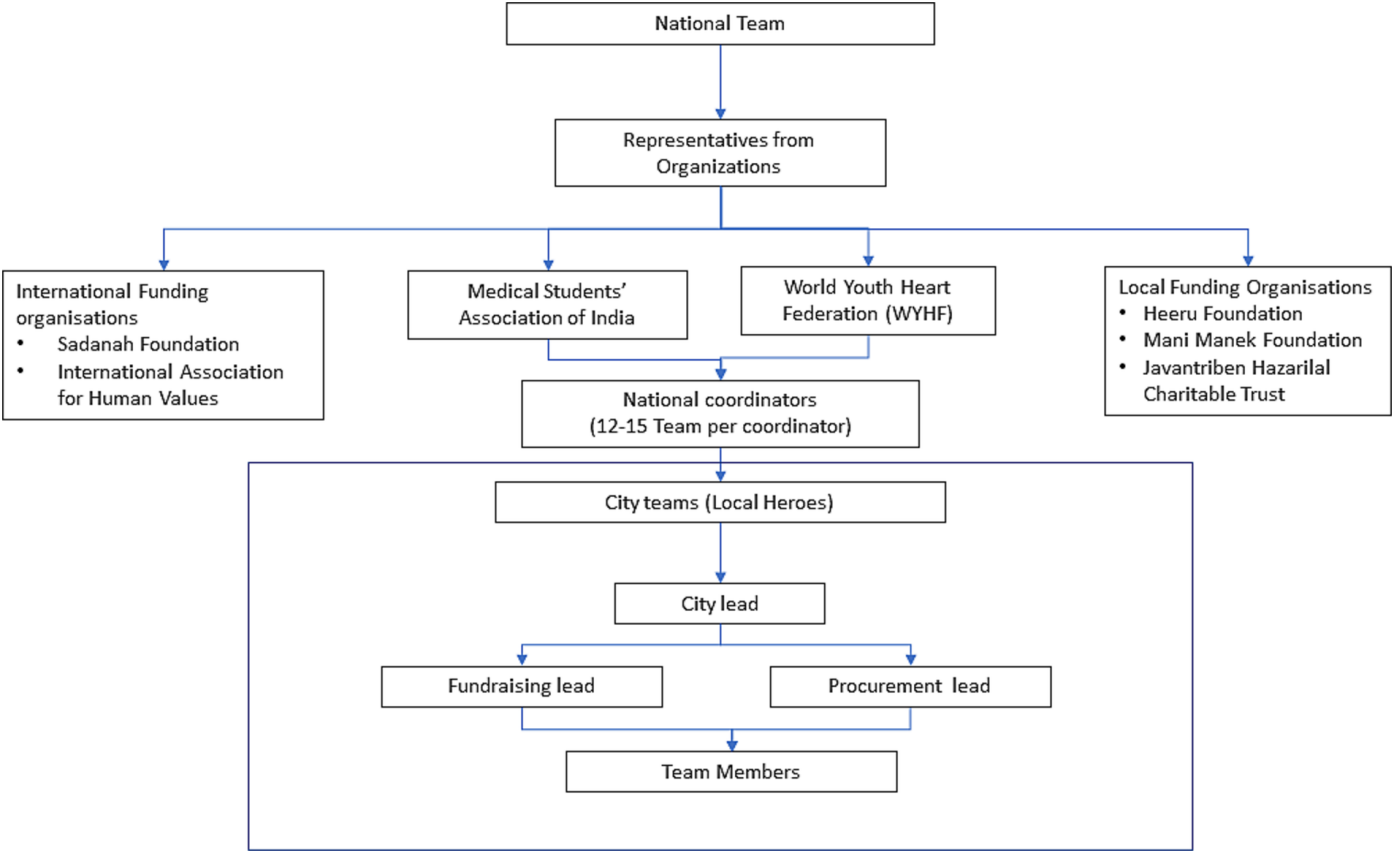
Team structure of The Local Heroes Project.

Based on learnings of the pilot project, a nationwide hyperlocal project was launched in more cities. During the scale up we collaborated with multiple International (Sadanah Foundation, International Association for Human Values), National (Medical Students’ Association of India) and Local (Heeru Foundation, Mani Manek Foundation and Javantriben Hazarilal Charitable Trust) stakeholders for the project. A national team was formed consisting of representatives from each partnering organization. This team oversaw the project and ensured national supply chain, national funding and volunteer recruitment continued. Capacity building efforts to recruit volunteers were put in place via awareness drives on Instagram, twitter and WhatsApp groups of the organizations. The national coordinators were responsible for twelve to fifteen city teams each. The city volunteers coordinated the progress with the city leads, who in turn communicated it to the national coordinators who reported to the national team (Figure 1).

Once a minimum of three volunteers joined the LHP of a city, a needs assessment at primary, secondary and tertiary centers was done. Simultaneously, they were also asked to start fundraising efforts by contacting local philanthropic organizations, community groups such as neighborhood associations, and community leaders. A message was circulated describing the project, the donations which were made during the LHP pilot in Vadodara, which served as a model for other cities, and transparent ways to donate. These included: (1) direct payment to the supplier and (2) donation via a fundraising app called Ketto. Each donor was sent a certificate thanking them for their donation and mentioning the amount donated. The final aspect of the LHP was supply chain management. The volunteers contacted medical supply stores in their city and neighboring cities on a rolling basis, as the needs assessment proceeded. If the supplies could not be ensured locally, a national supplier was contacted by the national team who ensured timely supply to the centers. All the three aspects of the LHP continued simultaneously and relied heavily on prompt communication among all the levels of the project. In the cities where volunteers were finding it difficult to fundraise, the national funds were mobilized as seed funding to donate the most essential supplies required by the centers. These national funds were a summation of the donations made on Ketto and funds raised internationally by the Sadanah Foundation, an organization that aims to increase sustainable access to healthcare in low resource settings by providing funding to grassroot organizations. A follow up message was circulated on the previously established channels to donors to make the second attempt more credible by showing them local donations. A snowballing effect was seen where more fundraising was possible when the donors saw credible pre-existing donations. When observed retrospectively, seed funding was required in the majority of the cities.

Internal deliberations with all the volunteers from Vadodara and suggestions from community medicine residents were taken into consideration. Seed funding was used to increase credibility, which resulted in snowballing of funds in the cities. This model is based on four pillars: (1) Replicability, (2) Transparency, (3) Diversity, and (4) Consent. LHP was remote, online and relied on tools such as Google Drive, WhatsApp and phone calls. The sheets used in the project could be used as templates, increasing the replicability of the project. All the needs assessment, donor and supplier information were accessible to the national team which consisted of representatives from all the organizations involved. Additional transparency was ensured by documenting the donations via pictures and receipts which were uploaded and stored on the same Google Drive. Central communication was in English while the local communication was both in English and local languages. Local teams helped tackle the cultural and linguistic barriers on ground. Consent was taken from all the hospitals, healthcare centers and donors informing them the data collected during the project would only be used for providing supplies and research which was indicated via a Google Forms checkbox.

### 2.2 Project monitoring and evaluation

The project was closely monitored by the national team. A channel of communication was established between the volunteers and the national leads on, facilitated through platforms such as WhatsApp and regular Zoom meetings. National coordinators had individual calls with respective city groups to brief the volunteers about the project and their roles. Weekly zoom meetings were held to provide the city leads with a platform to share their progress and challenges faced in addition to need based communication on WhatsApp. Every donation, every fundraising event and progress was documented with the help of Google Spreadsheets. The national team had regular calls which allowed us to address any obstacles along the way.

A Qualitative Comparative Analysis (QCA) model was adopted for the impact evaluation (8). The question of interest to us was, − “Can local fundraising and resource mobilization by local youth, who are directed by a national team, bridge the shortage of essential drugs and PPEs during an infectious disease pandemic?.” Outcome of the project was delivery of supplies in healthcare centers of each city. Each city was considered as an individual case for the analysis. Factors considered to have an influence on the outcome were – size of local teams, contacts and relations of local team members with funders, drugs and PPEs suppliers and local administration, reporting frequency of the local team to the national team, receptiveness of local authorities towards the donations, and number of health centers that required supplies. Factors were scored according to the crisp set scoring on a scale of 0–1, where 0 represented complete absence of the factor, 1 represented complete presence of the factors. This data was maintained in Google Spreadsheets and analyzed based on Truth Table Analysis using the fsQCA software. Findings were interpreted and presented in the results section.

## 3 Observations and impact

**Figure 2:**
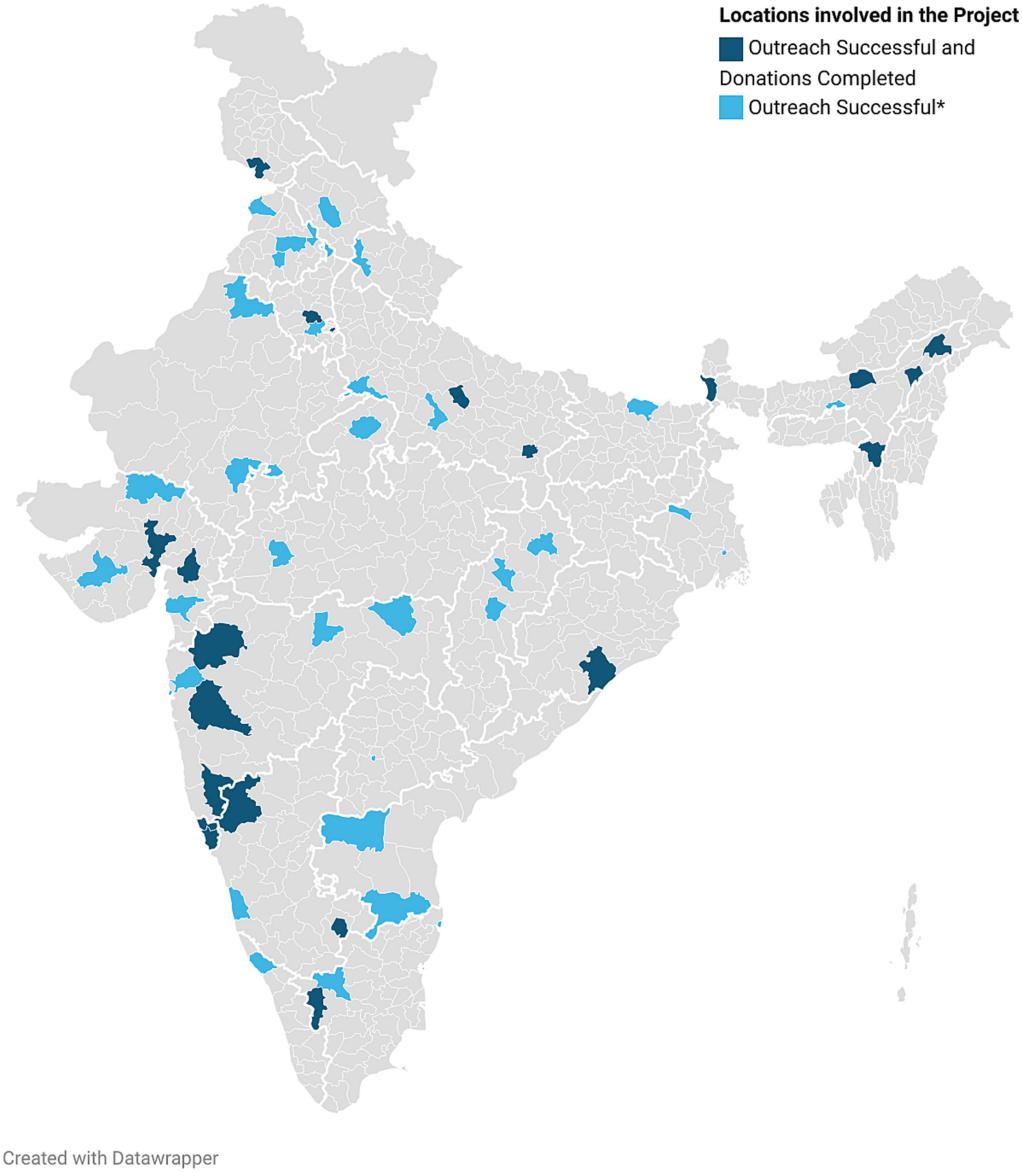
Locations in India where The Local Heroes Project was implemented. Name of the cities. Agra, Ahmedabad, Akola, Ambikapur, Amritsar, Anandpur sahib, Bahadurgarh, Bangalore, Barnala, Belgaum, Berhampur, Bilaspur, Chandigarh, Chennai, Coimbatore, Daman, Dehradun, Delhi NCR, Dibrugarh, Durgapur, Erode, Goa, Guwahati, Gwalior, Hanumangarh, Hyderabad, Indore, Jammu City, Jorhat, Kannaur, Kanpur, Kolhapur, Kolkata, Kurnool, Lucknow, Ludhiana, Madhubani, Mandi, Mumbai, Nagpur, Nashik, Nimbahera, Palanpur, Panchkula, Pondicherry, Pune, Raipur, Rajkot, Rohtak, Silchar, Siliguri, Surat, Tezpur, Thane, Tirupati, Udupi, Vadodara, Varanasi (Underlined – Donations completed).

The project had spread across India within the first 2 weeks including 58 city groups and 438 volunteers (Figure 2). 48.2% of the city groups completed needs assessment and 37.9% completed their donations. The rest of the city groups lacked sufficient volunteers and response to do the needs assessment. The cities which completed donations also received support from local organizations, school groups (in Assam), college groups (in Jammu, Assam and Belgaum), neighborhood associations (in New Delhi, Rohtak, Coimbatore, Lucknow), college departments, local politicians (in Rohtak and Ahmedabad), which were pivotal to carry out donations. A sense of giving back was observed in Anklav, Gujarat, where no pulse oximeters were previously available. Seven pulse oximeters were donated to the Khadol Primary Healthcare Centre, Anklav, allowing for effective monitoring of the patients. Post recovery, one of the patients of Khadol along with other individuals from their community donated INR 30,000 back to the initiative. A fulfillment of unmet needs and first-hand decrease in the unmet demand of essential supplies was observed. Out of 48 cities, twenty relied on a national supplier for all their needs, majority of which were from the eastern and southern states. Ten cities raised the money locally, another nine cities utilized seed funding while the remaining nine cities used both locally raised money and seed funding. All 48 Primary Health Care Centers in Agra and 40 Primary Health Care Centers in Ahmedabad were covered in a single donation drive. The fundraising and volunteer recruitment were aided by mention of our work on social media, various local and national print media as well as newspapers (9, 10).

Based on the Truth Table Analysis (Table 1) under QCA it was found that under complex solution, factors such as team strength more than four, local needs assessment, regular reporting during monthly meeting, receptive local administration, donation to more than 2 health centers and donation of supplies worth > = Rs 5,000 in each city (raw coverage 0.44, consistency 1) were more important contributors for success of the outcome, i.e., delivery of essential supplies in healthcare centers, to fulfill the unmet need. However, the parsimonious solution, i.e., the most important factor for success was found to be donation of supplies worth > = Rs 5,000 (raw coverage 1, consistency 1). This meant that the supply crisis in each city could only be met if funds more than Rs 5,000 could be raised for acquisition and donation of supplies. An account of location wise distribution of supplies along with their QCA scoring can be found in Table 2 (more details on data and analysis in Supplement 1, 2 respectively). The total amount of donation (supplies and monetary funds) summed up to about INR 2.45 million (USD 30,000). The cost of conducting the drive was INR 132,298, which was accounted as cost for shipments.

**Table 1:** Truth Table by QCA analysis.

Team creation (WhatsApp group created)	Team strength (>/= 4 people)	Local funding utilised	Needs assessment	Supplier contact (>/= 2 contacts)	Attended monthly team meetings	Receptive local admin (respond to WhatsApp messages within 72 h)	Donation (>/= INR 5,000 worth supplies)	Outreach (>/= 2 health centers)	Rural Center (>/= 1 rural PHC)	Frequncy	Outcome (supplies need fulfilled)	raw consist.	PRI consist.	SYM consist
1	1	1	1	1	1	1	1	1	1	7	1	1	1	1
1	1	0	1	0	1	1	1	1	1	2	1	1	1	1
1	0	0	1	0	0	1	1	0	0	1	1	1	1	1
1	0	1	1	0	1	1	1	0	0	1	1	1	1	1
1	1	1	1	1	1	1	1	0	0	1	1	1	1	1
1	1	1	1	0	0	0	1	1	0	1	1	1	1	1
1	1	1	1	1	1	1	1	1	0	1	1	1	1	1
1	0	0	1	1	1	0	1	0	1	1	1	1	1	1
1	0	0	1	0	0	1	1	0	1	1	1	1	1	1
1	1	0	1	0	0	1	1	0	1	1	1	1	1	1
1	0	0	1	0	1	1	1	0	1	1	1	1	1	1
1	1	0	1	0	1	1	1	0	1	1	1	1	1	1
1	0	1	1	0	1	1	1	0	1	1	1	1	1	1
1	1	0	1	1	1	1	1	0	1	1	1	1	1	1
1	0	1	1	1	1	1	1	0	1	1	1	1	1	1
1	0	1	1	0	1	1	1	1	1	1	1	1	1	1
1	1	1	1	0	1	1	1	1	1	1	1	1	1	1
1	1	0	1	1	1	1	1	1	1	1	1	1	1	1

**Table 2:** Factors contributing to the success of the project in each city as per QCA scoring.

Cities	Supplies need fulfilled	Team creation (WhatsApp group created)	Team strength (>/= 4 people)	Local funding utilised	Needs assessment	Supplier contact (>/= 2 contacts)	Attended monthly team meetings	Receptive local admin (respond to WhatsApp messages within 72 h)	Donation (>/= INR 5,000 worth supplies)	Outreach (>/= 2 health centers)	Rural center (>/= 1 rural PHC)
Jammu	1	1	1	1	1	1	1	1	1	1	0
Delhi	1	1	1	1	1	1	1	1	1	1	1
Vadodara	1	1	1	1	1	1	1	1	1	1	1
Surat	1	1	1	1	1	0	1	1	1	1	1
Ahmedabad	1	1	1	1	1	1	1	1	1	1	1
Kolhapur	1	1	1	0	1	0	1	1	1	1	1
Nashik	1	1	1	1	1	1	1	1	1	1	1
Pune	1	1	1	0	1	0	1	1	1	1	1
Agra	1	1	0	1	1	0	1	1	1	1	1
Varanasi	1	1	0	0	1	0	0	1	1	0	1
Lucknow	1	1	0	1	1	0	1	1	1	0	1
Silchar	1	1	1	0	1	0	1	1	1	0	0
Dibrugarh	1	1	1	1	1	1	1	1	1	0	1
Guwahati	1	1	0	0	1	1	1	0	1	0	0
Dhemaji	1	1	0	0	1	0	1	1	1	0	1
Tezpur	1	1	1	1	1	1	1	1	1	1	1
Jorhat	1	1	0	1	1	1	1	1	1	0	0
Siliguri	1	1	1	0	1	0	0	1	1	0	1
Coimbatore	1	1	0	1	1	0	1	1	1	0	1
Rohtak	1	1	1	1	1	1	1	1	1	1	1
Goa	1	1	1	0	1	1	1	1	1	1	1
Belgaum	1	1	1	1	1	1	1	1	1	1	1
Bangalore	1	1	1	0	1	1	1	1	1	0	1
Mumbai	1	1	1	1	1	0	0	0	1	1	1
Behrampur	1	1	0	0	1	0	0	1	1	0	0
Chennai	0	1	1	0	1	0	0	1	0	0	0
Erode	0	1	0	0	0	0	0	1	0	0	0
Pondicherry	0	1	0	0	0	0	0	0	0	0	0
Kurnool	0	1	0	0	0	0	0	0	0	0	0
Tirupati	0	1	0	0	0	0	0	0	0	0	0
Madhubani	0	1	1	0	0	0	0	1	0	0	0
Raipur	0	1	0	0	0	0	0	0	0	0	0
Bilaspur	0	1	0	0	0	0	0	0	0	0	0
Ambikapur	0	1	0	0	0	0	0	0	0	0	0
Daman	0	1	1	0	0	0	0	0	0	0	0
Rajkot	0	1	0	0	0	0	0	0	0	0	0
Panchkula	0	1	0	0	0	0	0	0	0	0	0
Bahadurgarh	0	1	0	0	0	0	0	0	0	0	0
Mandi	0	1	0	0	0	0	0	0	0	0	0
Udupi	0	1	0	0	0	0	0	0	0	0	0
Kannaur	0	1	0	0	0	0	0	0	0	0	0
Nagpur	0	1	0	0	0	0	0	0	0	0	0
Indore	0	1	0	0	0	0	0	0	0	0	0
Gwalior	0	1	0	0	0	0	0	0	0	0	0
Ludhiana	0	1	1	0	0	0	0	1	0	0	0
Chandigarh	0	1	0	0	0	0	0	0	0	0	0
Barnala	0	1	0	0	0	0	0	0	0	0	0
Amritsar	0	1	0	0	0	0	0	0	0	0	0
Anandpur Sahib	0	1	0	0	0	0	0	0	0	0	0
Nimbahera	0	1	0	0	0	0	0	0	0	0	0
Hanumangarh	0	1	0	0	0	0	0	0	0	0	0
Hyderabad	0	1	0	0	0	0	0	0	0	0	0
Durgapur	0	1	0	0	0	0	0	0	0	0	0
Kolkata	0	1	0	0	0	0	0	0	0	0	0
Dehradun	0	1	0	0	0	0	0	0	0	0	0
Kanpur	0	1	0	0	0	0	0	0	0	0	0

## 4 Lessons learnt

We learnt several essential lessons from the field. Lessons from our case study enlisted below, can be utilized to plan and implement fundraiser campaigns for public health interventions in future.

1. Efficient Team Recruitment and Deployment: the national and local teams were swiftly assembled in under a month, leveraging the existing organizational infrastructure. This rapid response highlights the need to redirect the existing resources in times of crisis.
2. Empowering Community Leaders through Hyperlocal Models: our experience illuminated the power of addressing crises through a hyperlocal approach. By identifying, stratifying, and empowering community leaders, we enabled them to take action with minimal external assistance. This approach fosters a sense of belonging and shared purpose within the community to mobilize effective change from within. Operating through online media also decreases capital invested in such a model.
3. Co-Creation with Stakeholders: we recognized the value of involving on-ground healthcare workers, hospital administrators, and patients in the development of interventions. Collaborating with these key stakeholders ensures that our initiatives are well-informed and tailored to the specific needs and challenges faced by the local healthcare ecosystem.
4. The Role of Collaboration: our observations underscored the importance of collaboration for the sustainability of hyperlocal models. External support, often at the national or international level, acts as a safety net when local efforts fall short. It’s worth noting that these external resources can also kickstart local initiatives, as exemplified by the transformation of initial seed funds into a locally generated funding stream. Furthermore, our experience emphasized the necessity of involving a diverse group of individuals of the community through intersectoral collaboration. Our collaboration with other existing non-profits provided combined resources to execute the project.
5. Optimizing the Global Supply Chain: proactive communication with national suppliers early in the project proved pivotal in ensuring the availability of essential supplies, especially in regions where shortages were prevalent. Notably, international shipping channels were leveraged to source some critical items, such as ventilators. The establishment of a national fund further bridged fundraising gaps at the local level, ensuring a more robust supply chain.
6. Sustainability and Replicability: we recognized the importance of establishing a dedicated impact assessment team that operates concurrently with the project’s execution. This approach enables us to thoroughly document the project’s progress and outcomes, allowing for a comprehensive impact assessment once the crisis response phase concludes. This robust documentation is essential for assessing the project’s sustainability and replicability in similar scenarios.

Our primary drivers were making a crisis relief model which was replicable, easy to understand and relied on the accountability of people living there locally. The project was intersectional that involved volunteers of various demographics. This ensured diversity in the volunteer population that helped the project adapt to the diverse communities of India. Moreover, we effectively utilized the offline work mode for successful donations while complying with social distancing rules. During this period, we worked with individuals we had never met, thousands of kilometers away. Volunteers who were not healthcare workers learnt critical global health skills involved in a pandemic response.

There were also a few barriers to our project. Transportation of essential supplies due to the nationwide lockdown posed a key barrier. This made the implementation of the hyperlocal model paramount. Although external funds were necessary in many cities, the donations could only have been carried out with the help of the communities affected. When external aid manages to empower local communities and leaders, the impact is multifold. Additionally, there were cities where volunteers could not complete their needs assessment due to their inability to travel during the lockdown. Moreover, due to factors such as an urgency to develop a model and ensuring timely donations, the impact assessment aspect of the project could not develop at the same pace as the rest of the project.

## 5 Strengths and limitations of evaluation

The main strength of QCA lies in its focus on in-depth cases, combined with an ability to draw out patterns across many different cases. QCA does have some limitations that need to be recognised. The scoring process for factors can require quite complex judgements, and there is a risk that scoring can become too subjective. This is particularly true of crisp-set QCA which effectively divides all factors into “yes-no” answers.

The cost–benefit and social return on investment could not be calculated due to the following reasons. Firstly, due to the overwhelming number of cases and relative shortage of healthcare workers (Healthcare Workers), the receiving hospitals could not keep a record of the number of patients and Healthcare Workers benefited through our supplies. Secondly, there is no record of how long the resources donated lasted and given the diversity of resources, it varies considerably. Resources like Personalized Protective Equipments and masks were used once whereas oximeters and ventilators have a utility of years. Lastly, the use of the resources overlapped. For example, masks were used by the same Healthcare Workers who used sanitizers. Hence, calculating the impact of individual donations remains a challenge. Our current efforts are directed at calculating the impact of this project with the help of collaborators who specialize in health economics.

## 6 Conclusion

The second wave of COVID 19 pandemic in India significantly increased the burden on the healthcare system and resulted in shortage of health resources. The crisis was further intensified by misallocation of resources, the WYHF tackled this problem in solidarity under the LHP. The gaps in resource allocation were identified by a baseline assessment followed by targeted donations. The project was executed by multidisciplinary teams led by youth leaders. LHP serves as a model for tackling acute, large-scale crises with teams working remotely, utilizing tools such as social media, online drives and cell phones. The hyperlocal model with local volunteers working on fundraising, supply chain management and donations enhanced the efficiency. This project also provides a structured method for analyzing the project impact. Co-creation, structured implementation science efforts and teamwork are essential to make this project replicable.

## Data availability statement

The original contributions presented in the study are included in the article/Supplementary material, further inquiries can be directed to the corresponding author.

## Author contributions

DP: Conceptualization, Data curation, Investigation, Methodology, Project administration, Resources, Supervision, Writing – original draft, Writing – review & editing. PSh: Conceptualization, Investigation, Methodology, Project administration, Resources, Supervision, Writing – review & editing. SR: Data curation, Formal analysis, Methodology, Visualization, Writing – original draft, Writing – review & editing. AA: Data curation, Formal analysis, Methodology, Writing – original draft, Writing – review & editing. DS: Investigation, Methodology, Project administration, Writing – review & editing. PSa: Investigation, Methodology, Project administration, Writing – review & editing. KG: Investigation, Methodology, Project administration, Writing – review & editing. MD: Investigation, Methodology, Project administration, Writing – review & editing. YT: Investigation, Methodology, Project administration Writing – review & editing. SP: Investigation, Methodology, Project administration, Writing – review & editing. ZZ: Methodology, Project administration, Resources, Supervision, Writing – review & editing. MS: Methodology, Project administration, Resources, Supervision, Writing – review & editing.

## References

[1] FRONTLINE T. 2021: COVID-19 second wave [Internet]. (2022). Available at: https://frontline.thehindu.com/the-nation/public-health/india-at-75-epochal-moments-2021-covid-19-second-wave/article65732713.ece (Accessed April 9, 2023).

[2] Choudhary OP Priyanka SI Rodriguez-Morales AJ. Second wave of COVID-19 in India: dissection of the causes and lessons learnt. Travel Med Infect Dis. (2021) 43:102126. doi: 10.1016/j.tmaid.2021.102126, PMID: 34144178 PMC8214078

[3] Asrani P Eapen MS Hassan MI Sohal SS. Implications of the second wave of COVID-19 in India. Lancet Respir Med. (2021) 9:e93–4. doi: 10.1016/S2213-2600(21)00312-X, PMID: 34216547 PMC8245060

[4] Agarwala P Bhargava A Gahwai DK Negi SS Shukla P Dayama S. Epidemiological characteristics of the covid-19 pandemic during the first and second waves in Chhattisgarh, Central India: a comparative analysis. Cureus. (2022) 14:e24131. doi: 10.7759/cureus.24131 35573570 PMC9106595

[5] Kapoor M Panda PK. India’s second covid wave: how is it different from the first wave? Int J Infect Dis. (2022) 116:S50. doi: 10.1016/j.ijid.2021.12.121

[6] Ghosh S Moledina N Hasan MM Jain S Ghosh A. Colossal challenges to healthcare workers combating the second wave of coronavirus disease 2019 (COVID-19) in India. Infect Control Hosp Epidemiol 43:1–2. Available at: https://www.ncbi.nlm.nih.gov/pmc/articles/PMC8193204/10.1017/ice.2021.257 PMC8193204 34075871

[7] Batheja D Kurian V Buteau S Joy N Nair A. Role of oxygenation devices in alleviating the oxygen crisis in India. PLOS Glob Public Health. (2023) 3:e0002297. doi: 10.1371/journal.pgph.0002297, PMID: 37590175 PMC10434891

[8] Schatz F Welle K (2016). ‘Qualitative comparative analysis: a valuable approach to add to the evaluator’s toolbox? Lessons from recent applications.’ CDI Practice Paper No 13, 2016.

[9] Kaushik M. These youngsters are bridging the urban-rural healthcare divide. Forbes India Available at: https://www.forbesindia.com/article/covid19-frontline-warriors/these-youngsters-are-bridging-the-urbanrural-healthcare-divide/68499/1

[10] Rupera Prashant. Gujarat: Junior doctors go beyond call of Covid duty: https://t.co/H4HM5xa3r6?amp=1

