# Supplementary material for: The Local Heroes Project: a youth-led pan-India hyperlocal crisis relief model during the COVID-19 pandemic
Source: Front Public Health. 2023 Dec 7;11:1282289. doi: 10.3389/fpubh.2023.1282289 (PMC10740211; doi:10.3389/fpubh.2023.1282289)
Supplement: Supplementary file 1 [file Table_1.docx]

**Calculations for Return of Investment**

**1. Financial Input**

Input by donations: INR 24,49,964.89

Input in volunteer hours: INR 3,88,377.6 + INR 3,13,689.6 = INR 702.067.2

*Calculations:*

1. Average duration of work by local team from needs assessment to donation:

| Needs assessment | 2 days |
| --- | --- |
| Seeking donations | 5 days |
| Ordering supplies, delivering it to centers | 5 days |
| Average volunteer work per day | 1 hr |
| Total number of volunteer hours per city per volunteer | 12 hours |

- Number of volunteers: 208
- Total volunteer hours of all cities (1): 208 x 12 = 2496 hours = 332.8 working days
- Minimum wage of graduates (2): INR 32750 per month = INR 1167 per day
- Cost of volunteering in LHP: 332.8 x 1167 = INR 3,88,377.6

1. Average duration of work by national team from beginning of LHP till the last donation:

Duration of Local heroes Project: from 1st May to 30th July (12 weeks):

Number of hours per day = 3 hours

Number of people in the national team = National leads: 4 medical students ; WYHF Board: 4 medical interns

- Volunteer hours (1): 84 x 3 x 8 = 2016 hours = 268.8 working days
- Minimum wage of graduates (2) : INR 32750 per month = INR 1167 per day
- Cost of volunteering in LHP: 268.8 x 1167 = INR 3,13,689.6

1. Cost of shipping and transportation for donations: INR 132,298
2. Cost of shipping and transportation for donations along with salary of volunteers: INR 834,365

**Qualitative Comparative Analysis Model^4^**

-- COMPLEX SOLUTION ---

frequency cutoff: 1

consistency cutoff: 1

| Factor combinations | raw coverage | unique coverage | consistency |
| --- | --- | --- | --- |
| TeamCreationWhatsappgroupcreated*~LocalFundingutilised*NeedsAssesment*~SupplierContact>/=2contacts*ReceptiveLocalAdminrespondtowhatsappmessageswithin72hrs*Donation>/=INR5,000worthsupplies*~Outreach>/=2healthcenters*RuralCenter>/=1ruralPHC | 0.16 | 0.08 | 1 |
| TeamCreationWhatsappgroupcreated*TeamStrength>/=4people*~LocalFundingutilised*NeedsAssesment*Attendedmonthlyteammeetings*ReceptiveLocalAdminrespondtowhatsappmessageswithin72hrs*Donation>/=INR5,000worthsupplies*RuralCenter>/=1ruralPHC | 0.20 | 0.04 | 1 |
| TeamCreationWhatsappgroupcreated*TeamStrength>/=4people*NeedsAssesment*Attendedmonthlyteammeetings*ReceptiveLocalAdminrespondtowhatsappmessageswithin72hrs*Donation>/=INR5,000worthsupplies*Outreach>/=2healthcenters*RuralCenter>/=1ruralPHC | 0.44 | 0.28 | 1 |
| TeamCreationWhatsappgroupcreated*~TeamStrength>/=4people*~LocalFundingutilised*NeedsAssesment*~SupplierContact>/=2contacts*~Attendedmonthlyteammeetings*ReceptiveLocalAdminrespondtowhatsappmessageswithin72hrs*Donation>/=INR5,000worthsupplies*~Outreach>/=2healthcenters | 0.08 | 0.04 | 1 |
| TeamCreationWhatsappgroupcreated*~TeamStrength>/=4people*LocalFundingutilised*NeedsAssesment*~SupplierContact>/=2contacts*Attendedmonthlyteammeetings*ReceptiveLocalAdminrespondtowhatsappmessageswithin72hrs*Donation>/=INR5,000worthsupplies*~Outreach>/=2healthcenters | 0.08 | 0.04 | 1 |
| TeamCreationWhatsappgroupcreated*~TeamStrength>/=4people*LocalFundingutilised*NeedsAssesment*Attendedmonthlyteammeetings*ReceptiveLocalAdminrespondtowhatsappmessageswithin72hrs*Donation>/=INR5,000worthsupplies*~Outreach>/=2healthcenters*RuralCenter>/=1ruralPHC | 0.08 | 0.04 | 1 |
| TeamCreationWhatsappgroupcreated*TeamStrength>/=4people*LocalFundingutilised*NeedsAssesment*SupplierContact>/=2contacts*Attendedmonthlyteammeetings*ReceptiveLocalAdminrespondtowhatsappmessageswithin72hrs*Donation>/=INR5,000worthsupplies*~RuralCenter>/=1ruralPHCTeamCreationWhatsappgroupcreated*LocalFundingutilised*NeedsAssesment*~SupplierContact>/=2contacts*Attendedmonthlyteammeetings*ReceptiveLocalAdminrespondtowhatsappmessageswithin72hrs*Donation>/=INR5,000worthsupplies*Outreach>/=2healthcenters*RuralCenter>/=1ruralPHC | 0.08 | 0.08 | 1 |
| TeamCreationWhatsappgroupcreated*LocalFundingutilised*NeedsAssesment*~SupplierContact>/=2contacts*Attendedmonthlyteammeetings*ReceptiveLocalAdminrespondtowhatsappmessageswithin72hrs*Donation>/=INR5,000worthsupplies*Outreach>/=2healthcenters*RuralCenter>/=1ruralPH | 0.08 | 0.04 | 1 |
| TeamCreationWhatsappgroupcreated*TeamStrength>/=4people*LocalFundingutilised*NeedsAssesment*~SupplierContact>/=2contacts*~Attendedmonthlyteammeetings*~ReceptiveLocalAdminrespondtowhatsappmessageswithin72hrs*Donation>/=INR5,000worthsupplies*Outreach>/=2healthcenters*~RuralCenter>/=1ruralPHC | 0.04 | 0.04 | 1 |
| TeamCreationWhatsappgroupcreated*~TeamStrength>/=4people*~LocalFundingutilised*NeedsAssesment*SupplierContact>/=2contacts*Attendedmonthlyteammeetings*~ReceptiveLocalAdminrespondtowhatsappmessageswithin72hrs*Donation>/=INR5,000worthsupplies*~Outreach>/=2healthcenters*RuralCenter>/=1ruralPHC | 0.04 | 0.04 | 1 |

solution coverage: 1

solution consistency: 1

Cases with greater than 0.5 membership in term TeamCreationWhatsappgroupcreated*~LocalFundingutilised*NeedsAssesment*~SupplierContact>/=2contacts*ReceptiveLocalAdminrespondtowhatsappmessageswithin72hrs*Donation>/=INR5,000worthsupplies*~Outreach>/=2healthcenters*RuralCenter>/=1ruralPHC: Varanasi (1,1),

Silchar (1,1), Dhemaji (1,1), Siliguri (1,1)

Cases with greater than 0.5 membership in term TeamCreationWhatsappgroupcreated*TeamStrength>/=4people*~LocalFundingutilised*NeedsAssesment*Attendedmonthlyteammeetings*ReceptiveLocalAdminrespondtowhatsappmessageswithin72hrs*Donation>/=INR5,000worthsupplies*RuralCenter>/=1ruralPHC: Kolhapur (1,1),

Pune (1,1), Silchar (1,1), Goa (1,1),

Bangalore (1,1)

Cases with greater than 0.5 membership in term TeamCreationWhatsappgroupcreated*TeamStrength>/=4people*NeedsAssesment*Attendedmonthlyteammeetings*ReceptiveLocalAdminrespondtowhatsappmessageswithin72hrs*Donation>/=INR5,000worthsupplies*Outreach>/=2healthcenters*RuralCenter>/=1ruralPHC: Jammu (1,1),

Delhi (1,1), Vadodara (1,1), Surat (1,1),

Ahmedabad (1,1), Kolhapur (1,1), Nashik (1,1),

Pune (1,1), Rohtak (1,1), Goa (1,1),

Belgaum (1,1)

Cases with greater than 0.5 membership in term TeamCreationWhatsappgroupcreated*~TeamStrength>/=4people*~LocalFundingutilised*NeedsAssesment*~SupplierContact>/=2contacts*~Attendedmonthlyteammeetings*ReceptiveLocalAdminrespondtowhatsappmessageswithin72hrs*Donation>/=INR5,000worthsupplies*~Outreach>/=2healthcenters: Varanasi (1,1),

Behrampur(Orissa) (1,1)

Cases with greater than 0.5 membership in term TeamCreationWhatsappgroupcreated*~TeamStrength>/=4people*LocalFundingutilised*NeedsAssesment*~SupplierContact>/=2contacts*Attendedmonthlyteammeetings*ReceptiveLocalAdminrespondtowhatsappmessageswithin72hrs*Donation>/=INR5,000worthsupplies*~Outreach>/=2healthcenters: Lucknow (1,1),

Coimbatore (1,1)

Cases with greater than 0.5 membership in term TeamCreationWhatsappgroupcreated*~TeamStrength>/=4people*LocalFundingutilised*NeedsAssesment*Attendedmonthlyteammeetings*ReceptiveLocalAdminrespondtowhatsappmessageswithin72hrs*Donation>/=INR5,000worthsupplies*~Outreach>/=2healthcenters*RuralCenter>/=1ruralPHC: Jorhat (1,1),

Coimbatore (1,1)

Cases with greater than 0.5 membership in term TeamCreationWhatsappgroupcreated*TeamStrength>/=4people*LocalFundingutilised*NeedsAssesment*SupplierContact>/=2contacts*Attendedmonthlyteammeetings*ReceptiveLocalAdminrespondtowhatsappmessageswithin72hrs*Donation>/=INR5,000worthsupplies*~RuralCenter>/=1ruralPHC: Dibrugarh (1,1),

Tezpur (1,1)

Cases with greater than 0.5 membership in term TeamCreationWhatsappgroupcreated*LocalFundingutilised*NeedsAssesment*~SupplierContact>/=2contacts*Attendedmonthlyteammeetings*ReceptiveLocalAdminrespondtowhatsappmessageswithin72hrs*Donation>/=INR5,000worthsupplies*Outreach>/=2healthcenters*RuralCenter>/=1ruralPHC: Surat (1,1),

Agra (1,1)

Cases with greater than 0.5 membership in term TeamCreationWhatsappgroupcreated*TeamStrength>/=4people*LocalFundingutilised*NeedsAssesment*~SupplierContact>/=2contacts*~Attendedmonthlyteammeetings*~ReceptiveLocalAdminrespondtowhatsappmessageswithin72hrs*Donation>/=INR5,000worthsupplies*Outreach>/=2healthcenters*~RuralCenter>/=1ruralPHC: Mumbai (1,1)

Cases with greater than 0.5 membership in term TeamCreationWhatsappgroupcreated*~TeamStrength>/=4people*~LocalFundingutilised*NeedsAssesment*SupplierContact>/=2contacts*Attendedmonthlyteammeetings*~ReceptiveLocalAdminrespondtowhatsappmessageswithin72hrs*Donation>/=INR5,000worthsupplies*~Outreach>/=2healthcenters*RuralCenter>/=1ruralPHC: Guwahati (1,1)

--- PARSIMONIOUS SOLUTION ---

frequency cutoff: 1

consistency cutoff: 1

| Factor combinations | raw coverage | unique coverage | consistency |
| --- | --- | --- | --- |
| Donation>/=INR5,000worthsupplies | 1 | 1 | 1 |

solution coverage: 1

solution consistency: 1

Cases with greater than 0.5 membership in term Donation>/=INR5,000worthsupplies: Jammu (1,1),

Delhi (1,1), Vadodara (1,1), Surat (1,1),

Ahmedabad (1,1), Kolhapur (1,1), Nashik (1,1),

Pune (1,1), Agra (1,1), Varanasi (1,1),

Lucknow (1,1), Silchar (1,1), Dibrugarh (1,1),

Guwahati (1,1), Dhemaji (1,1), Tezpur (1,1),

Jorhat (1,1), Siliguri (1,1), Coimbatore (1,1),

Rohtak (1,1), Goa (1,1), Belgaum (1,1),

Bangalore (1,1), Mumbai (1,1), Behrampur(Orissa) (1,1)

*References*

1. Holiday policy & hours of work [Internet]. [cited 2023 Apr 9]. Available from: <https://documents.doptcirculars.nic.in/D2/D02est/jcm3.html>
2. DU pay band categories: <http://www.du.ac.in/du/uploads/rti/Annexure-XII.pdf>
3. Bodycomb A, Del Baglivo M. Using an automated tool to calculate return on investment and cost benefit figures for resources: the Health Sciences and Human Services Library experience. J Med Libr Assoc. 2012 Apr;100(2):127-30. doi: 10.3163/1536-5050.100.2.011. PMID: 22514509; PMCID: PMC3324795.
